# 6-Bromoindole-3-acetonitrile Attenuates DSS-Induced Colitis by Inhibiting Epithelial Cell Pyroptosis

**DOI:** 10.3390/foods15101697

**Published:** 2026-05-12

**Authors:** Da Hong, Ximing Yang, Zhihui Chang, Lushun Yuan, Ming Du, Shuzhen Cheng

**Affiliations:** 1 State Key Laboratory of Marine Food Processing & Safety Control, School of Food Science and Technology, Dalian Polytechnic University, Dalian 116034, China; 2 National Engineering Research Center of Seafood, School of Food Science and Technology, Dalian Polytechnic University, Dalian 116034, China; 3 Department of Vascular Surgery, Intervention Center, Shanghai General Hospital, Shanghai Jiao Tong University School of Medicine, Shanghai 201620, China; 4 College of Food Science and Engineering, Dalian Ocean University, Dalian 116023, China

**Keywords:** 6-bromoindole-3-acetonitrile, Ulcerative colitis, pyroptosis, intestinal barrier, myeloperoxidase (MPO)

## Abstract

Ulcerative colitis is a chronic inflammatory bowel disease that requires new treatment approaches beyond traditional anti-inflammatory drugs. In this study, we analyzed publicly available single-cell RNA sequencing data from a DSS-induced colitis mouse model and identified pyroptosis as a key biological process linked to epithelial damage. Based on this, we screened marine-derived brominated indoles for potential pyroptosis inhibitors and identified 6-bromoindole-3-acetonitrile as a promising candidate. Our results show that this compound significantly alleviates DSS-induced colitis in mice, with notable body weight recovery and a drop in Disease Activity Index (DAI) scores from about 8.5 to below 4 (*p* < 0.05). At the molecular level, it lowers the mRNA levels of *Nlrp3*, *Caspase-1*, and other pyroptosis-related genes, indicating suppression of the pyroptotic pathway. Moreover, treatment helps restore the intestinal barrier by supporting goblet cell regeneration and strengthening tight junctions. Molecular docking suggests that 6-bromoindole-3-acetonitrile binds stably to the active site of myeloperoxidase (MPO), with a binding energy of −18.1 kcal/mol, offering a possible structural basis for its anti-inflammatory effects. Together, these findings point to a marine-derived compound that reduces both inflammation and pyroptosis, representing a promising strategy for treating ulcerative colitis. Notably, these results come from preclinical studies and need further validation in clinical settings.

## 1. Introduction

Ulcerative colitis (UC) is a chronic, relapsing form of inflammatory bowel disease (IBD) characterized by debilitating symptoms and a complex pathogenesis, which together present a substantial clinical challenge. The condition clinically manifests as diffuse inflammation of the colonic mucosa, resulting in symptoms such as persistent diarrhea, rectal bleeding, and abdominal pain that together substantially compromise patient quality of life [1]. While the etiology of UC involves a dysregulated immune response, compromised intestinal barrier, and microbial dysbiosis, a complete understanding of its drivers remains elusive, hampering the development of curative therapies [2].

Current management strategies for UC, including aminosalicylates, corticosteroids, and biologics, often fall short. While these treatments can induce remission, their clinical utility is often constrained by significant limitations. Clinical data indicate that up to 30% of UC patients do not respond to initial anti-TNF therapy, and among those who do, nearly 50% may lose response over time. Furthermore, long-term use of corticosteroids is associated with systemic adverse effects in over 50% of patients, while newer biologics still face high annual relapse rates ranging from 20% to 40% [3]. These quantitative challenges underscore the urgent need for safer, multi-target therapeutic alternatives [4]. There is a pressing need to develop novel therapeutic agents that are both safe and effective. In this context, natural products, especially those from underexplored sources like the marine environment, represent a promising avenue for drug discovery [5]. Marine organisms produce a wealth of unique chemical scaffolds with potent bioactivities, among which brominated indoles have garnered attention for their anti-inflammatory properties [6].

To pinpoint a critical pathological mechanism for intervention, we analyzed public single-cell RNA-seq data of a colitis mouse model. This methodology revealed the involvement of pyroptosis, a type of programmed cell death characterized by its highly inflammatory nature, as a pivotal process driving epithelial loss in UC [7]. Subsequent screening of marine compounds in a pyroptosis model revealed that 6-bromoindole-3-acetonitrile exhibited the most significant pyroptosis-inhibitory activity.

6-bromoindole-3-acetonitrile is a brominated indole alkaloid originally isolated from the marine sponge *Pseudosuberites hyalinus* (Phylum Porifera, Class Demospongiae), a species commonly found in temperate coastal ecosystems [8]. Its molecular structure possesses features that facilitate interactions with bioactive molecules. Its bromine atom enhances lipid solubility and cellular penetration, while its acetonitrile group provides a flexible site for structural optimization. Based on our initial findings, we hypothesize that 6-bromoindole-3-acetonitrile exerts its protective effect by modulating myeloperoxidase (MPO) activity, which subsequently suppresses the *NLRP3*-mediated pyroptotic cascade in colonic epithelial cells, thereby preserving the intestinal barrier.

Herein, we evaluate the therapeutic potential of 6-bromoindole-3-acetonitrile in a murine model of DSS-induced colitis, which recapitulates key features of human UC. We assess its effects on disease activity, inflammatory cytokine production, neutrophil infiltration, and intestinal barrier integrity. Furthermore, we mechanistically dissect its role in suppressing the NLRP3-mediated pyroptosis pathway and explore its interaction with myeloperoxidase (MPO) using molecular docking. This study aimed to evaluate the therapeutic efficacy of 6-bromoindole-3-acetonitrile in DSS-induced colitis, analyze cell-specific transcriptomic changes in colitis using public scRNA-seq data, and elucidate its inhibitory effects on the *NLRP3/Caspase-1* pyroptotic pathway.

## 2. Materials and Methods

### 2.1. Reagents and Materials

The Marine Natural Product Library (Cat. L6400) was commercially obtained from TargetMol Chemicals Inc. (Shanghai, China). 6-Bromoindole-3-acetonitrile (Cat. No. TN7198) was supplied by Topscience Co., Ltd. (Shanghai, China). Dextran sodium sulfate (DSS, 36,000–50,000 Da, Cat. No. 60316ES) was obtained from Yeasen Biotech Co., Ltd. (Shanghai, China). Lipopolysaccharide (LPS, Cat. No. ST1470) and Nigericin (Nig, Y261764) were sourced from Beyotime Biotechnology (Shanghai, China) and MedChemExpress (Shanghai, China), respectively.

Total RNA extraction was performed with the Eastep^®^ Super Total RNA Extraction Kit (Promega, Beijing, China). Subsequent cDNA synthesis was conducted using the FastKing gDNA Dispelling RT SuperMix, and quantitative PCR (qPCR) analyses were carried out with the SuperReal PreMix (both from Tiangen Biochemical Technology Co., Ltd., Beijing, China). Protein concentration was quantified with a BCA Protein Assay Kit (Beyotime Biotechnology, Shanghai, China). Myeloperoxidase (MPO) activity and the levels of cytokines, including *IL-1β*, *IL-10*, *IL-17*, and *IL-18*, were determined using specific commercial assay kits from the Nanjing Jiancheng Institute of Biological Engineering (Nanjing, China).

For immunofluorescence staining, the following antibodies were employed: primary antibodies against mouse Ly6G (rat monoclonal) and MUC-2 (rabbit monoclonal) from Wuhan Saiwei Biotech Co., Ltd. (Wuhan, China). Corresponding secondary antibodies included Alexa Fluor 594-conjugated goat anti-rat IgG and Alexa Fluor 488-conjugated goat anti-rabbit IgG, also acquired from Wuhan Saiwei Biotech Co., Ltd. (Wuhan, China).

### 2.2. Bioinformatics Analysis

The scRNA-seq dataset GSE148794 of colon tissues from the control group and the UC model group was obtained from the GEO database “https://www.ncbi.nlm.nih.gov/geo/ (accessed on 3 November 2025)”. The workflow began with stringent quality control. Cells with gene counts between 500 and 4000 and UMI counts below 15,000 were selected to exclude empty droplets and potential doublets [9], while the mitochondrial gene expression limit of <10% was applied to remove apoptotic or damaged cells. These thresholds are consistent with established standards for murine colonic tissue to ensure high-quality transcriptomic data for downstream analysis. Subsequently, the decontX algorithm was applied to remove ambient RNA contamination [10], and DoubletFinder (version 2.0.4) was used to exclude doublets, ensuring data purity. To address potential batch effects across different samples, the Harmony algorithm was employed for integration and correction. The ‘Sample’ identity was used as the batch variable to regress out technical variations while preserving biological differences between the control and colitis groups. The clustering resolution was determined as 0.2 through multi-resolution testing (Figure S1). The current study mainly focused on epithelial cell subpopulations, thereby enabling a comparative analysis of differentially expressed genes between the DSS-induced colitis model and the healthy control group. We performed Gene Set Enrichment Analysis (GSEA) to profile pathway activity using our custom pan-cell death gene collection.

### 2.3. Cellular Pyroptosis Assay

Human colonic epithelial cells (NCM460) were maintained in DMEM containing 10% (*v*/*v*) heat-inactivated fetal bovine serum and 1% (*v*/*v*) penicillin–streptomycin (100 U/mL penicillin and 100 μg/mL streptomycin). Cells were plated in 96-well plates at a density of 1 × 10^4^ cells per well and allowed to adhere for 24 h. To induce pyroptosis, cells were first stimulated with 1 μg/mL LPS for 4 h, followed by treatment with 10 μM nigericin for 1 h. Following successful model establishment, the cells were pretreated with a library of 144 marine-derived compounds for 24 h, which included 6-bromoindole-3-acetonitrile at concentrations of 10, 50, or 100 μM. Untreated cells served as the positive control group.

While PI staining cannot exclusively identify pyroptosis because it stains any cells with impaired membrane integrity, we selected this approach for preliminary compound screening due to its high efficiency, real-time observability, and simple operation. PI can quickly reflect the membrane pore formation triggered by GSDMD during pyroptosis, which aligns with widely used experimental procedures in the field [11]. After the respective treatments, cells were incubated with propidium iodide (PI) staining solution (50 μg/mL) in the dark for 15–30 min. Fluorescence intensity was measured using a Tecan Infinite M200 Pro microplate reader (Männedorf, Switzerland) at excitation/emission wavelengths of 535 nm and 617 nm. All cellular experiments were performed with at least three independent biological replicates (*n* = 3) to ensure statistical robustness. PI uptake was used as an indicator of pyroptotic cell death, as it enters cells through damaged plasma membranes and intercalates with nucleic acids.

### 2.4. DSS-Induced Mice Colitis Model and 6-Bromoindole-3-acetonitrile Treatment

Male C57BL/6 mice (8 weeks old, 22 ± 2 g) were sourced from Liaoning Changsheng Biotechnology Co., Ltd. (Shenyang, China). The animals were maintained under specific pathogen-free (SPF) conditions with a controlled temperature of 23 ± 1 °C, relative humidity of 50% ± 5%, and a 12/12 h light–dark cycle (lights on at 8:00 AM). Throughout the study, all mice had free access to standard rodent diet and autoclaved water. The experimental procedures were reviewed and approved by the Animal Experimental Ethics Committee of Dalian Polytechnic University (Approval No. DLPU2024085) and were performed in accordance with the institutional guidelines for the ethical care and use of laboratory animals.

Following a 7-day acclimation period, mice were randomly assigned to five groups (*n* = 9 per group) using a computer-generated random number sequence (GraphPad Prism 9.5.1). The control group (C) received normal drinking water along with daily intragastric administration of sterile saline (vehicle) for 14 days. In contrast, the DSS model group (MOD) was administered 3% (*w*/*v*) DSS in their drinking water for the first 7 days to induce colitis, followed by a return to normal water for the subsequent 7 days. Daily saline gavage was maintained throughout the entire 14-day period in the MOD group. For the low-dose treatment group (L), DSS-induced mice were given daily intragastric gavage of 6-Bromoindole-3-acetonitrile at 10 mg/kg body weight for 14 days, whereas the medium-dose treatment group (M) received the same compound at 50 mg/kg body weight via daily intragastric gavage for 14 days, and the high-dose treatment group (H) was administered 6-Bromoindole-3-acetonitrile at 100 mg/kg body weight through daily intragastric gavage over the 14-day duration. Fresh DSS solution was prepared daily to prevent degradation. Body weight was recorded daily to monitor disease progression. The doses of 6-Bromoindole-3-acetonitrile (10, 50, and 100 mg/kg) were selected based on preliminary dose-finding studies and the effective dose ranges reported for structurally similar indole derivatives in murine colitis models [12]. To minimize bias, the investigators were blinded to the treatment groups during the daily clinical assessments (body weight and stool monitoring) and the subsequent histological scoring and biochemical analyses. The group assignments were only unblinded after the completion of all data collection and statistical analyses.

### 2.5. Assessment of Disease Activity Index (DAI)

The DAI was evaluated by the percentage of weight loss, fecal consistency and fecal occult blood status of mice. The assessment criteria and specific classification rules are shown in Table S1 [13].

### 2.6. Tissue Collection

On day 14 of treatment, mice were fasted for 24 h (with free access to water) and sacrificed by cervical dislocation. The entire colon and spleen were harvested immediately. Colons were flushed with chilled, sterile phosphate-buffered saline (PBS) to clear luminal contents. A standard ruler was used to determine colon length. Documentation was achieved through digital imaging. Spleens were excised and weighed for determination of the spleen index, a recognized marker of systemic inflammatory status. For subsequent analyses, colonic segments were allocated into three portions: one preserved in 10% neutral buffered formalin for histology, another in 4% paraformaldehyde for immunofluorescence, and a third portion that was rapidly frozen in liquid nitrogen and maintained at −80 °C for molecular and biochemical investigations.

### 2.7. Histological and Immunofluorescence Staining

Formalin-fixed colon samples were processed through a graded ethanol series (70%, 80%, 90%, 95%, 100%), cleared in xylene, and embedded in paraffin. Sections of 5 μm thickness were cut, dewaxed, and rehydrated through a descending alcohol series. These sections were then subjected to hematoxylin and eosin (H&E) staining, involving 5 min hematoxylin and 1 min eosin incubation, followed by dehydration, clearing, and mounting in neutral balsam. Colon morphology and mucus layer thickness were evaluated on H&E-stained sections cut in different orientations using an Olympus BX53 light microscope (Tokyo, Japan).

Goblet cells were quantified by Alcian Blue/Periodic Acid–Schiff (AB-PAS) staining. Following the standard dewaxing and rehydration procedure, sections were treated with Alcian Blue (pH 2.5) for 30 min, rinsed thoroughly, subjected to oxidation with periodic acid, and finally stained with Schiff’s reagent. Following hematoxylin counterstaining, slides were dehydrated, cleared, and mounted. Quantification of goblet cells per crypt was performed by analyzing five randomly selected microscopic fields from each tissue section using ImageJ software (version 1.8.0, NIH, Bethesda, MD, USA).

For immunofluorescence detection of Ly6G (neutrophils) and MUC-2 (mucin), colon tissues were fixed in 4% paraformaldehyde, embedded in OCT compound, and stored at −80 °C. Frozen sections (5–10 μm) were air-dried, permeabilized (for MUC-2 staining only) with 0.1% Triton X-100, and blocked with 1% BSA. Sections were incubated overnight at 4 °C with primary antibodies (Ly6G 1:200; MUC-2 1:500), followed by appropriate Alexa Fluor-conjugated secondary antibodies (1:1000) for 1 h at room temperature. Nuclei were stained with DAPI, and slides were imaged on a Zeiss LSM 880 confocal microscope (Jena, Germany). Fluorescence intensity was quantified in five random fields per section using ImageJ. The fluorescence intensity and positive area were quantified using ImageJ software. Specifically, the ‘Threshold’ function was applied to each image to distinguish the target signal from the background. A consistent threshold range (determined by the ‘Auto-Threshold’ algorithm, ‘Default’ method) was established based on the negative control group and then applied uniformly to all experimental groups to ensure unbiased quantification of MUC-2 and Ly6G expression.

### 2.8. Inflammatory Stimulation Assay

NCM460 cells were seeded in 6-well plates at a density of 5 × 10^4^ cells/well and allowed to adhere for 24 h. The pyroptosis model was stimulated with 1 μg/mL LPS for 4 h and 10μM Nig for 1 h to activate the NLRP3 inflammasome, followed by 6-Bromoindole-3-acetonitrile (10, 50, or 100 μM) for 24 h. Cells were harvested to extract RNA (qRT-PCR) and determine expression levels immediately after treatment.

### 2.9. Quantitative Real-Time PCR (qRT-PCR)

Total RNA isolation from frozen colon tissues and cultured cells was carried out following the manufacturer’s guidelines for the Eastep^®^ Super Total RNA Extraction Kit. The concentration and purity of the extracted RNA were assessed on a Thermo Fisher Scientific NanoDrop 2000 micro-spectrophotometer (Waltham, MA, USA), and only specimens demonstrating an A260/A280 ratio within the 1.8–2.0 range were advanced to subsequent procedures. For cDNA synthesis, the FastKing gDNA Dispelling RT SuperMix was employed to reverse transcribe 1 μg of total RNA. Quantitative real-time PCR reactions were assembled in a total volume of 20 μL, comprising 10 μL of SuperReal PreMix, 0.4 μL each of forward and reverse primers, 2 μL of cDNA template, and 7.2 μL of nuclease-free water. Amplification was conducted on a QuantStudio 3 Real-Time PCR System using a thermal profile that included an initial denaturation at 95 °C for 15 min, followed by 40 cycles of denaturation at 95 °C for 10 s and combined annealing/extension at 60 °C for 30 s. Target gene expression levels were normalized against the endogenous control GAPDH, and relative transcript quantification was determined using the 2^−ΔΔCt^ method. Corresponding primer sequences are provided in Supplementary Tables S2 and S3.

### 2.10. Myeloperoxidase (MPO) Activity Assay

Frozen colon tissues were homogenized on ice using a tissue homogenizer (Servicebio SB-5900DTD, Wuhan, China) in the MPO assay buffer provided with the kit. The homogenates were then centrifuged at 12,000× *g* for 15 min at 4 °C to collect the supernatant. The total protein concentration in the supernatant was quantified to normalize MPO activity. Following the kit instructions, 50 µL of supernatant was combined with 150 µL of assay buffer, and the change in absorbance at 460 nm was monitored over 5 min using a microplate reader. MPO activity was calculated and expressed as units per milligram of protein (U/mg), where one unit is defined as the amount of enzyme that oxidizes 1 μmol of H_2_O_2_ per minute at 37 °C.

### 2.11. Enzyme-Linked Immunosorbent Assay (ELISA)

Colon tissue homogenates were subjected to centrifugation at 12,000× *g* for 15 min at 4 °C to obtain supernatant fractions. Cytokine concentrations (IL-1β, IL-10, IL-17, IL-18, and TNF-α) in the collected supernatants were measured with commercially available ELISA kits following the manufacturers’ recommended procedures. The assay procedure consisted of the following steps: initially, 100 μL of either standard solutions or sample supernatants were transferred to antibody-precoated microplate wells and maintained at 37 °C for 90 min. After five successive washes with washing buffer, 100 μL of biotin-conjugated detection antibody was applied to each well and incubated at 37 °C for 60 min. Following an additional washing cycle, 100 μL of streptavidin–horseradish peroxidase (HRP) conjugate was introduced and allowed to incubate for 30 min at 37 °C. Subsequently, after a final washing procedure, 100 μL of TMB substrate was introduced, and the plate was protected from light during a 15 min development period. The enzymatic reaction was stopped through addition of 50 μL of 2 M sulfuric acid, and optical density measurements were recorded at 450 nm using a microplate reader. Cytokine concentrations were calculated by reference to standard curves generated concurrently and normalized to total protein content, with results expressed as picograms per milligram of protein (pg/mg protein).

### 2.12. Molecular Docking

The initial MPO structure (PDB: 1CXP) was obtained from the RCSB Protein Data Bank “https://www.rcsb.org/ (accessed on 5 December 2025)“. The protein structure was prepared in Discovery Studio 2019 R2 (Biovia, San Diego, CA, USA) by removing water molecules and heteroatoms, followed by hydrogen atom addition. Energy minimization was then implemented using the CHARMm force field to achieve an optimized conformation. For the ligand, the 3D structure of 6-Bromoindole-3-acetonitrile (PubChem CID: 10169476) was acquired and converted to mol2 format. An identical preparation procedure—hydrogen addition and CHARMm-based geometry optimization—was applied to the ligand structure.

Molecular docking simulations were executed employing the CDOCKER protocol within Discovery Studio 2019 R2. The MPO active site was specified as a 10 Å radius sphere encompassing the heme cofactor, which constitutes the enzyme’s primary catalytic center. Ligand conformers were generated through random torsional sampling, with 10 poses docked per ligand. Binding affinity was assessed based on two key metrics: CDOCKER energy (reflecting total binding energy) and CDOCKER interaction energy (representing specific intermolecular interaction energy). The molecular forces, encompassing an array of noncovalent forces such as van der Waals, π-π stacking, hydrogen bonding, π-alkyl, and π-cation interactions, between 6-Bromoindole-3-acetonitrile and MPO were analyzed and visualized using Discovery Studio.

### 2.13. Statistical Analyses

All data were analyzed using GraphPad Prism 9.5.1 software (GraphPad Software Inc., San Diego, CA, USA) and are presented as the mean ± standard deviation (SD) with *n* = 6 biological replicates per group. Prior to parametric analysis, the normality of the data distribution was assessed using the Shapiro–Wilk test, and the homogeneity of variances was confirmed using Levene’s test. Statistical significance was determined using one-way analysis of variance (ANOVA) followed by Tukey’s post hoc test for multiple comparisons. A *p*-value < 0.05 was considered statistically significant (* *p* < 0.05, ** *p* < 0.01, *** *p* < 0.001). For bioinformatics analyses, we utilized RStudio (v4.2.0) in conjunction with several key packages: Seurat (version 4.4.0) for single-cell RNA sequencing data processing, ggplot2 (version 4.0.0) for data visualization, Cell types were manually annotated based on canonical marker genes, DoubletFinder (version 2.0.4) was used to identify and remove doublets, and fgsea (version 1.30.0) was applied to perform functional gene set enrichment analysis.

## 3. Results

### 3.1. Bioinformatics Analysis and Drug Screening

**Figure 1:**
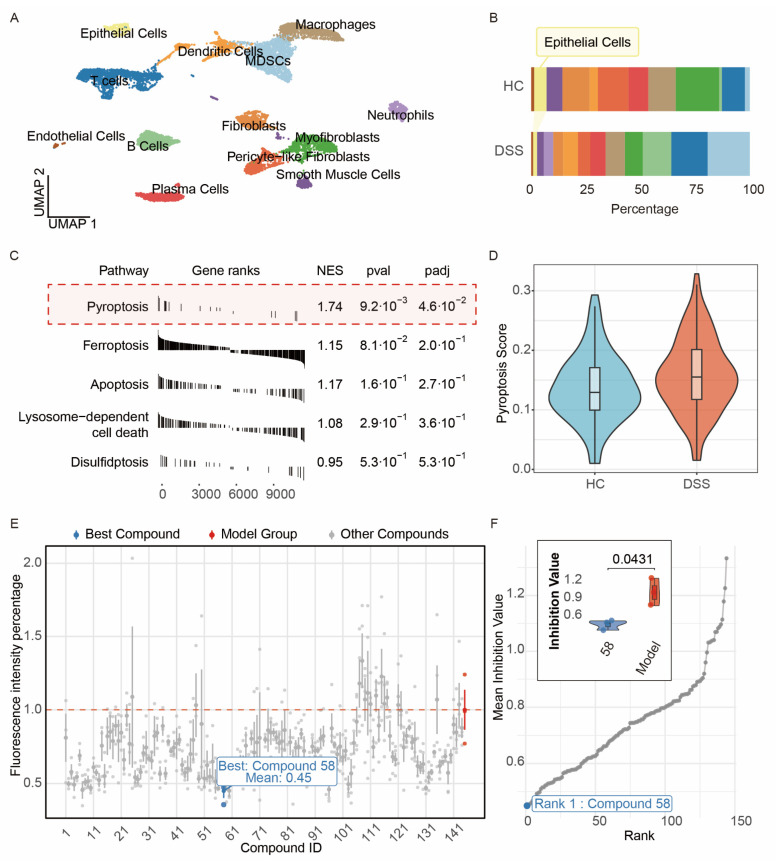
Single-cell RNA sequencing analysis reveals changes in colitis-associated cell populations and screening of marine-derived compounds targeting pyroptosis. (**A**) UMAP visualization of the single-cell transcriptomic landscape. Annotated cell types are listed; (**B**) Visualization of the proportion of each cell type in the normal group versus the DSS model group; (**C**) Pathway enrichment analysis via GSEA identifies pathways associated with the reduction in epithelial cells; (**D**) Pyroptosis pathway scores in the normal group versus the model group; (**E**) Quantitative fluorescence intensity values of propidium iodide (PI) staining for 144 marine-derived compounds in the LPS-induced pyroptosis model of NCM460 epithelial cells; (**F**) Ranking of compound inhibitory activity identified Compound **58** as the top candidate. Data are presented as mean ± SD from the indicated number of independent experiments, analyzed via one-way ANOVA (*n* = 3).

The raw single-cell data were processed using RStudio, and systematic quality control was performed on the raw single-cell RNA sequencing data. First, cells with a high proportion of mitochondrial gene expression were excluded to eliminate potential mitochondrial transcript contamination associated with cell damage or death [9]. Second, low-quality cells with fewer than 500 detected genes were filtered out. The DoubletFinder algorithm was subsequently applied to identify and remove potential doublets. Quality-controlled cells and their complete transcriptomes were retained for subsequent analysis. During the clustering analysis phase, the study focused on a subset of genes characteristic of colonic tissue. The “ScaleData” function was used to standardize the gene expression matrix while regressing out technical heterogeneity related to mitochondrial gene expression. We first integrated all data and identified 13 distinct cell clusters based on gene expression profiles, as shown in Figure 1A. Biological annotation of the cell clusters was performed by evaluating the expression patterns of the top ten canonical cell-type marker genes within each cluster (Figure S1).

To quantify population differences, the percentage distribution of different cell types across individual mice within each experimental group was calculated (Figure 1B). The results revealed a significant change in the proportion of epithelial cells, prompting subsequent in-depth analysis to focus on this cellular subset. Differential gene expression in epithelial cells between the Normal and DSS groups was identified using the “FindMarkers” function in the Seurat platform, followed by functional enrichment analysis using the fgsea algorithm concerning in-house pan cell death gene sets [14]. As shown in Figure 1C, Pyroptosis within the red box exhibited the highest NES value. Accordingly, pyroptosis was identified as the key mechanism underlying the reduction in epithelial cells. Finally, pyroptosis activity scores for each epithelial cell were calculated using the AUCell method, and the variation in pyroptosis activity between the Normal and DSS groups was visualized (Figure 1D), indicating that DSS-induced colitis promotes pyroptosis in colonic epithelial cells. Based on this finding, a high-throughput drug screen was conducted to evaluate the inhibitory effects of 144 marine compounds (Table S4) on pyroptosis. Following propidium iodide (PI) staining (Figure 1E), 6-Bromoindole-3-acetonitrile (compound #**58**) exhibited the lowest fluorescence intensity, indicating a significant inhibitory effect on pyroptosis. Consequently, this compound was selected as a candidate for subsequent mechanistic studies.

### 3.2. 6-Bromoindole-3-acetonitrile Ameliorate Disease progression in DSS-Induced Colitis

**Figure 2:**
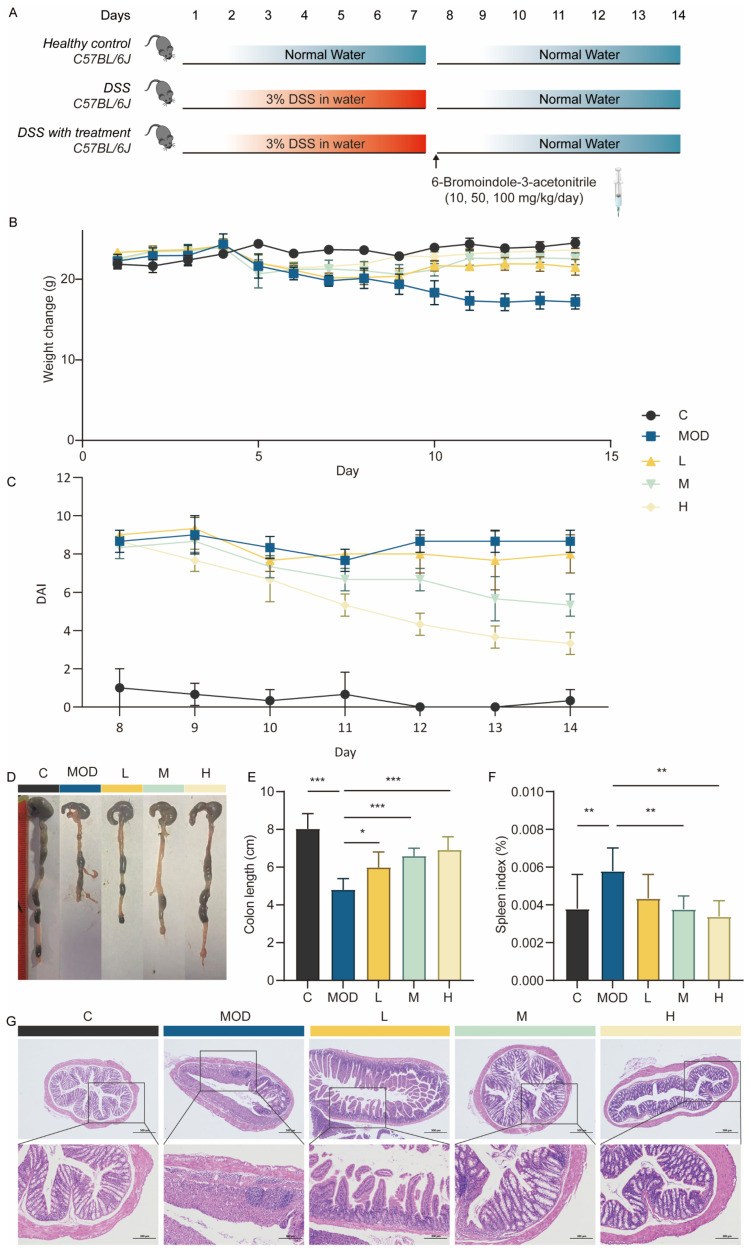
6-Bromoindole-3-acetonitrile mitigates the progression of DSS-induced colitis. (**A**) Schematic of the experimental design; (**B**) Dynamic changes in mouse body weight; (**C**) Mouse DAI Score (**D**) Representative images of colon length; (**E**) Quantified colon length; (**F**) Effect of 6-Bromoindole-3-acetonitrile on the spleen index; (**G**) Representative hematoxylin and eosin (H&E)-stained colon sections. Data are presented as mean ± SD from the indicated number of independent experiments, analyzed via one-way ANOVA. * *p* < 0.05, ** *p* < 0.01, *** *p* < 0.001 versus the DSS-treated group. (*n* = 6) (C: normal control group; MOD: colitis model group; L: low-dose 6-Bromoindole-3-acetonitrile; M: medium-dose; H: high-dose).

Experiments were conducted to evaluate the therapeutic effect of 6-Bromoindole-3-acetonitrile on dextran sulfate sodium (DSS)-induced colitis in mice. Following the induction of ulcerative colitis (UC) in mice by administration of 3% DSS for 7 days, 6-Bromoindole-3-acetonitrile was subsequently administered to the mice via oral gavage at varying doses from day 7 to day 14 (Figure 2A). Body weight trajectories of the mice are presented in Figure 2B. It was observed that the normal control (C) group maintained a progressive increase in body weight. In stark contrast, all other groups subjected to DSS intervention showed a significant reduction in body weight starting on day 4. The untreated model (MOD) group displayed the greatest degree of weight loss, thereby validating the successful replication of the ulcerative colitis model through DSS exposure. Compared with the MOD group, mice treated with L, M, and H doses of 6-Bromoindole-3-acetonitrile exhibited a significant reduction in weight loss, indicating that 6-Bromoindole-3-acetonitrile effectively mitigates DSS-induced colitis in a dose-dependent manner.

To evaluate the clinical efficacy of 6-bromoindole-3-acetonitrile, we monitored the Disease Activity Index (DAI) from day 8 to day 14 (Figure 2C). While the DAI in the 3% DSS-induced MOD group remained persistently high (8–9), the control group maintained a healthy score near 0. Notably, 6-bromoindole-3-acetonitrile treatment led to a significant, dose-dependent reduction in DAI scores beginning at days 9–10. By day 14, the score in the high-dose group dropped to below 4—a more than 50% improvement over the MOD group—demonstrating that the compound effectively alleviates clinical symptoms and promotes intestinal recovery. It has been documented that colon health is positively correlated with colon length [15]. As shown in Figure 2D,E, the colon length of DSS-induced colitis mice was markedly reduced compared with the control group (*p* < 0.01). By contrast, treatment at low-, medium-, and high-doses resulted in a dose-dependent recovery of colon length relative to the model group, with the high-dose group exhibiting the most substantial improvement (*p* < 0.01). The spleen plays a vital role in immune regulation, and inflammatory responses during colitis often induce splenomegaly along with an increase in spleen weight. Accordingly, the spleen index—calculated as the ratio of spleen mass to body weight—is widely used as a key parameter for assessing the severity of colitis [16]. Experimental results regarding the spleen index across different mouse groups are presented in Figure 2F. DSS-induced mice exhibited marked splenomegaly, whereas the spleen indices in the L, M, and H groups were significantly different from those in the MOD group (*p* < 0.05). These findings suggest that 6-Bromoindole-3-acetonitrile contributes to alleviating splenic damage, with efficacy enhanced as the concentration increases.

Colon sections from each experimental group were processed with hematoxylin and eosin (H&E) staining for histological evaluation, as presented in Figure 2G. A dose-responsive mitigation of colitis-associated histopathological damage was observed in the L, M, and H groups. Histological analysis of the MOD group showed near-complete loss of crypt architecture, extensive mucosal ulceration, and dense transmural inflammatory infiltration. In contrast, mice treated with 6-bromoindole-3-acetonitrile (100 mg/kg) exhibited remarkable preservation of the epithelial barrier, re-established crypt structures, and significantly reduced infiltration of immune cells into the lamina propria. These morphological improvements were consistently observed across all samples in the high-dose group, aligning with the observed recovery in colon length and DAI scores.

### 3.3. Repair of Colonic Mucosal Barrier in Mice upon 6-Bromoindole-3-acetonitrile Treatment

Goblet cells (GCs) are specialized secretory cells distributed in mucosal epithelial tissues. As the primary source of mucus, they are indispensable for upholding the structural and functional integrity of the mucosal barrier [17]. The mucosal epithelium is protected by a mucus layer, a gel-like barrier secreted onto its surface. Its thickness varies with tissue location, physiological state, and health status, and serves as a key indicator for evaluating mucosal barrier function. In an experimental colitis model established by dextran sodium sulfate (DSS) administration, the mucus layer is thinned, and GC-secreted mucus is reduced [18].

**Figure 3:**
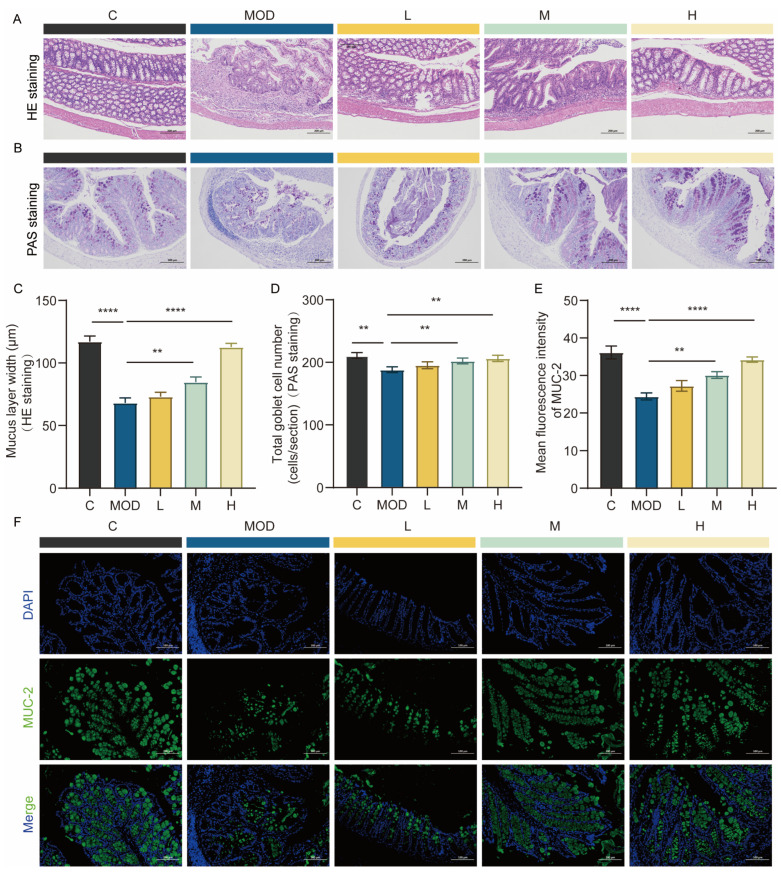
Repair of the colonic mucosal barrier in mice by 6-Bromoindole-3-acetonitrile. (**A**) Representative H&E-stained colon sections demonstrating mucus layer width. (**B**) Representative periodic acid–Schiff (PAS)-stained colon sections; (**C**) Quantification of colonic mucus layer thickness; (**D**) Quantification of goblet cell numbers; (**E**) Immunofluorescence staining of MUC-2 in colonic tissue; (**F**) Quantified MUC-2 protein expression levels following 6-Bromoindole-3-acetonitrile treatment. Data are presented as mean ± SD from the indicated number of independent experiments, analyzed via one-way ANOVA. ** *p* < 0.01, **** *p* < 0.0001 versus the DSS-treated group (*n* = 3) (C: normal control group; MOD: colitis model group; L: low-dose 6-Bromoindole-3-acetonitrile; M: medium-dose; H: high-dose).

In the DSS-induced colitis model, GCs are typically reduced due to inflammatory damage, resulting in thinning of the mucus layer and disruption of the intestinal barrier [19]. Compared with mice in the control (C) group, those in the DSS-induced (MOD) group exhibited a narrowed mucus layer (Figure 3A) and a decreased GC count (Figure 3B). In contrast, administration of 6-bromoindole-3-acetonitrile led to an increase in GC numbers and thickening of the mucus layer. Quantitative analysis revealed statistically significant differences (Figure 3C,D). These findings demonstrate that 6-bromoindole-3-acetonitrile can alleviate the pathological progression of colitis by restoring GC populations and enhancing the integrity of the mucus barrier. As established in prior studies, MUC-2 (the most abundant mucin secreted by GCs) maintains the integrity of the colonic mucus layer while protecting GCs themselves [20]. Compared with the MOD group, administration of 6-bromoindole-3-acetonitrile upregulated MUC-2 expression (Figure 3F), with a concentration-dependent significant increase observed (Figure 3E). Taken together, these findings collectively demonstrate that the therapeutic benefits of 6-bromoindole-3-acetonitrile against DSS-induced colitis are associated with its capacity to restore the integrity of the intestinal mucosal and epithelial barriers.

### 3.4. 6-Bromoindole-3-acetonitrile Maintains Intestinal Barrier Function by Enhancing Mucin Expression

To further elucidate the impact of 6-Bromoindole-3-acetonitrile on the colonic mucosal barrier, the expression levels of tight junction proteins and mucins were quantified. Tight junction proteins (TJs) are a class of proteins that structurally form and functionally maintain tight junctions. The intestinal epithelial barrier is primarily composed of a tightly continuous monolayer of intestinal epithelial cells (IECs), with TJs providing structural and functional support as a physical barrier to maintain mucosal homeostasis [21]. Impairment of tight junction barrier function is a key trigger and amplifier of colitis, and restoration of this function represents an important therapeutic goal. Mucins are a class of highly glycosylated macromolecular glycoproteins widely present on the surface of epithelial tissues in animals (e.g., respiratory tract, digestive tract, reproductive tract, and eyes) and constitute one of the main components of mucus. By forming a gel-like structure, mucins construct a physical and chemical barrier on the organism’s surface, exerting key physiological functions such as protection, lubrication, and defense.

**Figure 4:**
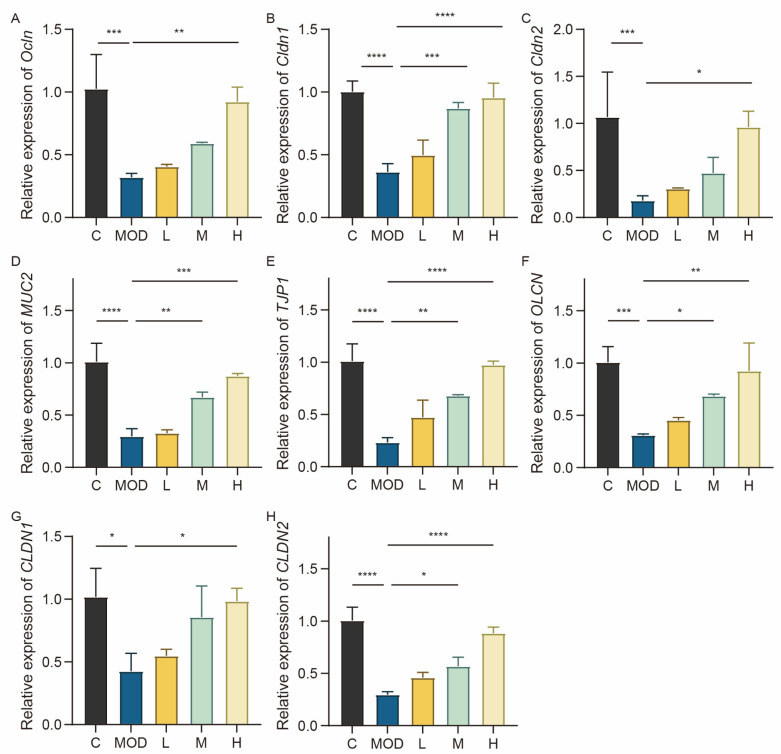
6-Bromoindole-3-acetonitrile maintains intestinal barrier function by enhancing mucin and tight junction protein expression. (**A**–**C**) mRNA expression levels of *Cldn1*, *Ocln*, and *Cldn2* in colonic tissue; (**D**–**H**) mRNA expression levels of occludin, *MUC2*, *CLDN1*, *TJP1*, and *CLDN2* in NCM460 epithelial cells. Data are presented as mean ± SD from the indicated number of independent experiments, analyzed via one-way ANOVA. * *p* < 0.05, ** *p* < 0.01, *** *p* < 0.001, **** *p* < 0.0001 versus the LPS-treated group and the DSS-treated group (*n* = 3) (C: normal control group; MOD: colitis model group; L: low-dose 6-Bromoindole-3-acetonitrile; M: medium-dose; H: high-dose).

In the DSS-induced (MOD) group, the expression levels of tight junction proteins (Cldn1, Cldn2, and Ocln) in mice were markedly reduced, whereas treatment with 6-bromoindole-3-acetonitrile restored the expression of these three proteins (Figure 4A–C), thereby contributing to the recovery of intestinal mucosal barrier integrity. In the lipopolysaccharide (LPS)-induced NCM460 cell inflammation model, compared with the LPS-induced MOD group, cells in the low-dose (L), medium-dose (M), and high-dose (H) 6-bromoindole-3-acetonitrile treatment groups exhibited significantly upregulated expression levels of tight junction proteins (*TJP1*, *OCLN*, *CLDN1*, and *CLDN2*) as well as the mucin *MUC2* (Figure 4D–H). This finding suggests that the compound can alleviate inflammatory damage by potentially enhancing the barrier function of intestinal epithelial cells, improving the integrity of tight junctions, and strengthening the protective effect of the mucus layer.

### 3.5. 6-Bromoindole-3-acetonitrile Alleviates DSS-Induced Colonic Inflammation

**Figure 5:**
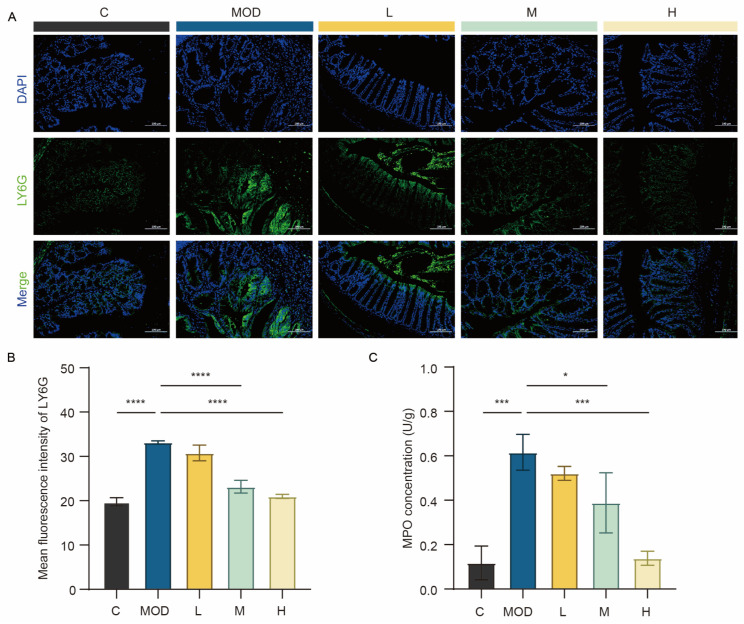
6-Bromoindole-3-acetonitrile alleviates DSS-induced colonic inflammation. (**A**) Immunofluorescence staining of Ly6g in colonic tissue; (**B**) Quantified Ly6g protein expression levels following 6-Bromoindole-3-acetonitrile treatment; (**C**) Quantified myeloperoxidase (MPO) activity/expression levels in colonic tissue. For all quantifications: Data are presented as mean ± SD from the indicated number of independent experiments, analyzed via one-way ANOVA. * *p* < 0.05, *** *p* < 0.001, **** *p* < 0.0001 versus the DSS-treated group (*n* = 3) (C: normal control group; MOD: colitis model group; L: low-dose 6-Bromoindole-3-acetonitrile; M: medium-dose; H: high-dose).

It has been reported that the presence of neutrophils is associated with the stage of anti-infection inflammation [22]. Therefore, neutrophils can be regarded as a key indicator for evaluating the severity of inflammation. Ly6G is mainly recognized as a specific marker of neutrophils [23]. As shown in Figure 5A, compared with the DSS-induced (MOD) group, the low-dose (L), medium-dose (M), and high-dose (H) 6-bromoindole-3-acetonitrile groups exhibited lighter neutrophil staining, indicating alleviated inflammation relative to the MOD group. The average fluorescence intensity of neutrophils (Figure 5B) revealed that the fluorescence intensity in the M and H groups was significantly decreased (*p* < 0.001). Myeloperoxidase (MPO) is a key marker of neutrophil activation and an indicator of oxidative stress in colitis [24]. The level of MPO (an oxidative stress indicator) in colon tissue is shown in Figure 5C. The significantly elevated MPO levels in the MOD group indicate that DSS-induced colitis leads to increased oxidative stress. Compared with the MOD group, treatment with 6-bromoindole-3-acetonitrile reduced MPO levels, suggesting a potential improvement in oxidative stress status.

### 3.6. 6-Bromoindole-3-acetonitrile Mitigates Inflammatory Cytokines Production

Cytokines are critical mediators in the cross-talk between the intestinal barrier and epithelial cells. A rise in intestinal permeability, however, can compromise the immune system’s ability to appropriately respond to gut bacterial antigens, often leading to the initiation of intestinal inflammation [25]. In the context of colitis, this dysfunction induces a hyperactivation of immune cells. This leads to an overabundance of pro-inflammatory cytokines that subsequently damage colonic tissues, ultimately driving disease pathology [26].

**Figure 6:**
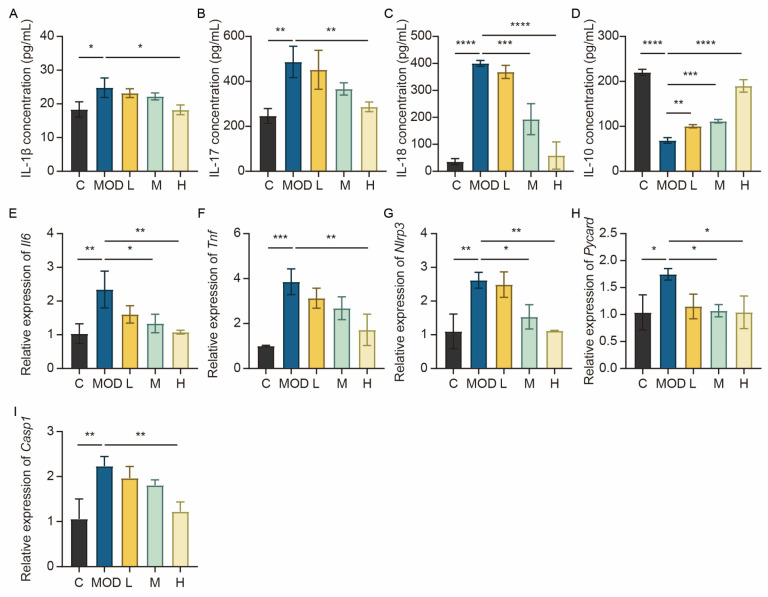
6-Bromoindole-3-acetonitrile mitigates DSS-induced colitis by modulating inflammatory cytokine expression. (**A**–**D**) Levels of *IL-18*, *IL-17*, *IL-1β*, and *IL-10* in colonic tissue, determined via enzyme-linked immunosorbent assay (ELISA); (**E**–**I**) mRNA expression levels of *IL-6*, *Tnf*, *Nlrp3*, *Pycard*, and *Casp1* in colonic tissue. Data are presented as mean ± SD from the indicated number of independent experiments, analyzed via one-way ANOVA. * *p* < 0.05, ** *p* < 0.01, *** *p* < 0.001, **** *p* < 0.0001 versus the DSS-treated group (*n* = 3) (C: normal control group; MOD: colitis model group; L: low-dose 6-Bromoindole-3-acetonitrile; M: medium-dose; H: high-dose).

To assess the anti-inflammatory properties of 6-bromoindole-3-acetonitrile, we quantified the levels of key cytokines (*IL-1β*, *IL-18*, *IL-17*, *IL-10*) in mouse colon homogenates via ELISA. *IL-17* is a key pro-inflammatory cytokine that plays a critical role in promoting intestinal mucosal injury, neutrophil infiltration, and inflammatory cascade amplification during colitis. As shown in Figure 6A–C, DSS induction (MOD group) significantly elevated pro-inflammatory cytokine levels compared to the normal control (C) (*p* < 0.01), indicating severe inflammatory damage. Conversely, treatment with medium (M) and high (H) doses of 6-bromoindole-3-acetonitrile significantly suppressed these elevations (*p* < 0.01) in a dose-dependent manner. Meanwhile, the anti-inflammatory cytokine IL-10 (Figure 6D), which was reduced in the MOD group, exhibited a dose-dependent recovery following treatment. qPCR analysis of colon tissue (Figure 6E–I) further confirmed these findings, showing that the mRNA levels of *IL-6*, *Tnf*, *Nlrp3*, *Pycard*, and *Casp1* were markedly upregulated in the MOD group but were progressively downregulated by 6-bromoindole-3-acetonitrile treatment, with higher doses yielding greater effects. Collectively, these results demonstrate that 6-bromoindole-3-acetonitrile alleviates colitis by rebalancing the inflammatory milieu, suppressing pro-inflammatory mediators while promoting anti-inflammatory responses.

### 3.7. 6-Bromoindole-3-acetonitrile Inhibits LPS-Induced Inflammation in NCM460 Epithelial Cells

**Figure 7:**
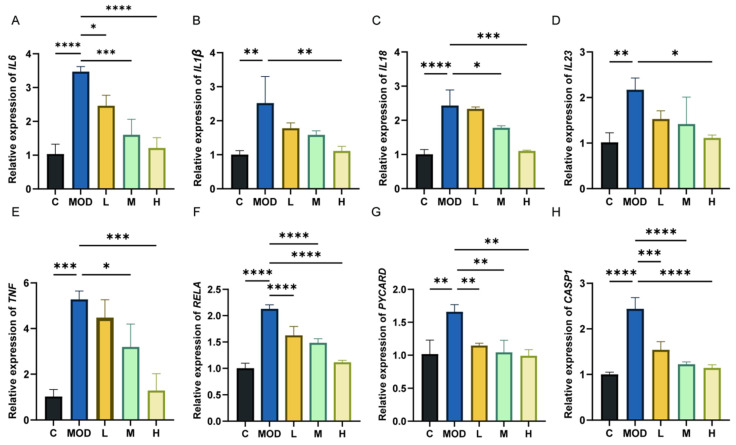
6-Bromoindole-3-acetonitrile inhibits LPS-induced inflammation in NCM460 epithelial cells. (**A**–**H**) mRNA expression levels of *IL-6*, *IL-1β*, *IL-18*, *IL-23*, *TNF*, *RELA*, *PYCARD*, and *CASP1* in NCM460 epithelial cells. Data are presented as mean ± SD from the indicated number of independent experiments, analyzed via one-way ANOVA. * *p* < 0.05, ** *p* < 0.01, *** *p* < 0.001, **** *p* < 0.0001 versus the LPS-treated group (*n* = 3) (C: normal control group; MOD: colitis model group; L: low-dose 6-Bromoindole-3-acetonitrile; M: medium-dose; H: high-dose).

As shown in Figure 7A–H, the mRNA levels of *IL-6*, *IL-1β*, *IL-23*, *IL-18*, *TNF*, *CASP1*, *PYCARD*, and *RELA* in LPS-induced NCM460 cells were measured by qPCR. Compared with the normal control (C) group, the mRNA levels of these inflammatory factors were significantly elevated in the LPS-induced (MOD) group. However, with the intervention of 6-bromoindole-3-acetonitrile, the mRNA levels of these inflammatory factors were significantly reduced. This trend is consistent with the changes in inflammatory factor mRNA expression observed in mouse colon tissues.

Furthermore, *Nlrp3* (detected in colonic tissues), *PYCARD*, and *CASP1* (detected in cells) are all core components of the pyroptosis pathway. Upon sensing danger signals, *NLRP3* recruits and activates Caspase-1 via the adaptor protein *PYCARD*; the activated *CASP1* then cleaves *GSDMD*, and the N-terminal fragment of *GSDMD* forms pores in the plasma membrane, ultimately leading to pyroptosis. The results demonstrate that 6-bromoindole-3-acetonitrile significantly reduces the mRNA expression levels of these key pyroptosis-related factors. This confirms that the compound alleviates DSS-induced colitis by potentially inhibiting the *NLRP3/PYCARD/CASP1* signaling axis and reducing pyroptosis in colonic epithelial cells.

### 3.8. Molecular Docking of MPO with 6-Bromoindole-3-acetonitrile

**Figure 8:**
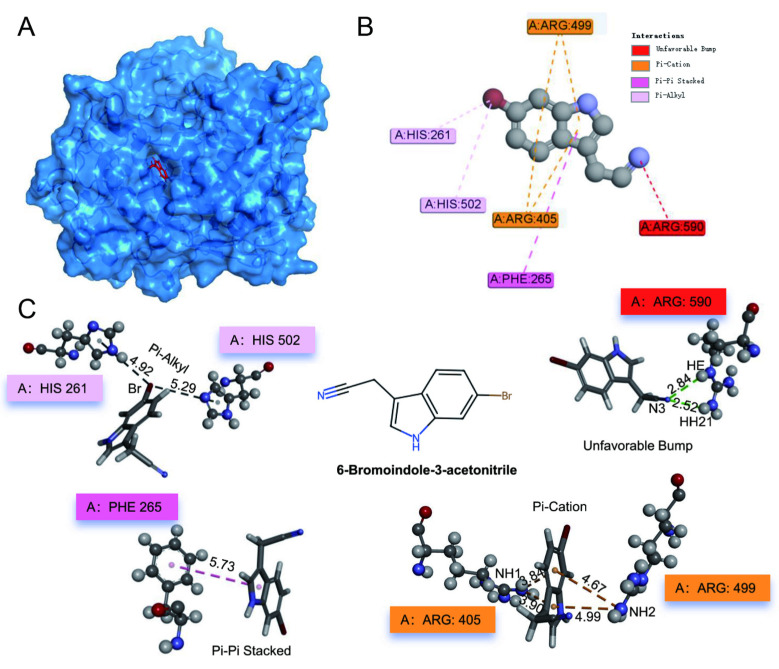
Docking conformation and chemical interactions of 6-Bromoindole-3-acetonitrile with MPO. (**A**) MPO-6-Bromoindole-3-acetonitrile complex. 6-Bromoindole-3-acetonitrile is shown as red sticks; (**B**) 2D interaction residues and interaction pattern diagram; (**C**) Key residues involved in the interaction between MPO and 6-Bromoindole-3-acetonitrile, along with a structural schematic of 6-Bromoindole-3-acetonitrile. Residues are represented using sphere representation. MPO residues are colored by atom type (C, H, O, and N atoms are shown in black, gray, red, and blue, respectively). Bonds formed between MPO and 6-Bromoindole-3-acetonitrile are indicated by dashed lines. Bond lengths are labeled adjacent to the bonds.

To elucidate the molecular basis for the pronounced therapeutic efficacy of 6-bromoindole-3-acetonitrile, molecular docking simulation was employed to investigate its interaction with myeloperoxidase (MPO). MPO is one of the most representative marker enzymes in neutrophils and plays a crucial role in innate immunity. With the rapid and continuous development of bioinformatics, bioinformatic methods have been widely applied in a series of studies using in silico tools [27]. The 3D docking mode and 2D interaction residues between MPO and 6-bromoindole-3-acetonitrile are shown in Figure 8A,B, respectively.

The binding potency was evaluated using the CDOCKER module, where two key energetic parameters—CDOCKER energy (representing the overall energy of the complex) and CDOCKER interaction energy (representing the non-bonded interaction energy between the ligand and the receptor)—were calculated. Generally, a more negative value (higher absolute value for the negative score) indicates a more thermodynamically favorable and stable interaction [28]. The CDOCKER interaction energy between 6-bromoindole-3-acetonitrile and MPO was −18.1 kcal/mol. Studies have shown that when the binding energy is less than −5 kcal/mol, the conformation of the ligand–protein complex remains stable during 100 ns molecular dynamics simulations [29]. However, it should be noted that this static value only represents a computational prediction of binding potential, rather than a definitive indicator of complex stability. These computational results suggest a favorable binding affinity between 6-bromoindole-3-acetonitrile and the MPO active site, providing a theoretical structural framework that aligns with its observed biological effects in alleviating colonic inflammation.

The chemical structure of 6-Bromoindole-3-acetonitrile and its interactions with amino acid residues are shown in Figure 8C. Further interaction analysis revealed that the binding between the ligand and the receptor is primarily mediated by multiple non-bonded interactions, including Conventional Hydrogen Bonds, Unfavorable Bumps, Pi-Cation interactions, Pi-Pi Stacked interactions, and Pi-Alkyl interactions. Specifically, an Unfavorable Bump was observed between the MPO residue ARG590 and the ligand; ARG405 and ARG499 formed stable Pi-Cation interactions with the aromatic ring of the ligand; PHE265 formed a Pi-Pi Stacked interaction with the ligand; and HIS261 and HIS502 formed Pi-Alkyl interactions with the bromine atom of the ligand. These multimodal, high-strength intermolecular interactions collectively contribute to the superior binding affinity of the complex.

As a key component of neutrophil granules, MPO contributes to host defense by catalyzing the generation of reactive halogen species (e.g., hypochlorous acid, HOCl) to eliminate pathogens. However, excessive MPO release leads to overproduction of HOCl, resulting in oxidative damage to host tissues and exacerbation of inflammatory responses. The findings of this study indicate that 6-bromoindole-3-acetonitrile efficiently binds to the active region of MPO, suggesting it may potentially modulate MPO enzymatic activity through competitive or allosteric inhibition mechanisms. This modulation would reduce the generation of harmful oxidative products and mitigate MPO-mediated tissue damage, thereby supporting the therapeutic effects of 6-bromoindole-3-acetonitrile in DSS-induced colitis models and providing novel molecular insights for the development of anti-inflammatory drugs.

## 4. Discussion

Our study provides compelling evidence that the marine-derived compound 6-Bromoindole-3-acetonitrile confers significant protection against DSS-induced colitis by concurrently targeting neutrophilic inflammation, *NLRP3*-mediated pyroptosis, and intestinal barrier dysfunction. This work bridges a critical gap by providing the first experimental evidence for the efficacy of this specific brominated indole in UC, underpinned by a rationally designed strategy that progressed from bioinformatic discovery to mechanistic validation.

Our investigation began with a bioinformatic analysis of the DSS-induced colitis model. This analysis revealed pyroptosis as a central mechanism, demonstrating that it critically drives epithelial cell loss—a key pathogenic event closely associated with the recurrence of ulcerative colitis [30]. This finding provided a compelling rationale for screening pyroptosis inhibitors. The subsequent in vitro screening of a marine compound library revealed 6-bromoindole-3-acetonitrile as a potent inhibitor, a result strongly corroborated in vivo by the compound’s significant suppression of the *NLRP3* inflammasome pathway (*NLRP3*, *PYCARD*, *Caspase-1*) and its effector cytokines (*IL-1β*, *IL-18*) [31]. The critical role of pyroptosis in amplifying inflammation through lytic cell death and the release of pro-inflammatory mediators [7] underscores the therapeutic relevance of targeting this pathway. Our data suggests the inhibition of pyroptosis as a central mechanism through which 6-bromoindole-3-acetonitrile exerts its protective effects.

Beyond modulating pyroptosis, the compound demonstrated a broad capacity to quell the inflammatory milieu. Treatment with 6-Bromoindole-3-acetonitrile significantly reduced Ly6G+ neutrophil infiltration and MPO activity, thereby effectively mitigating inflammatory responses. Neutrophils are among the first immune cells to infiltrate intestinal inflammatory sites, and their abundance serves as a reliable indicator of inflammation severity [32]. Myeloperoxidase (MPO), a secretory protein of neutrophils, is also a key marker of inflammatory burden [33]. The favorable binding affinity between 6-Bromoindole-3-acetonitrile and MPO, as predicted by molecular docking, provides a potential theoretical structural basis for this inhibition, and may suggest a plausible structural basis for its interaction with MPO, which could contribute to the observed reduction in enzymatic activity. Additionally, the compound favorably altered the cytokine balance, notably elevating the anti-inflammatory *IL-10* while suppressing pro-inflammatory signals such as *TNF-α*, *IL-6*, and *IL-17* [34].

Recent studies have shown that bioactive substances derived from marine and aquatic organisms, such as oyster-derived peptide–zinc complexes, can improve intestinal barrier function and restore mitochondrial homeostasis via dual absorption pathways, thereby enhancing nutrient bioavailability and alleviating intestinal injury [35]. In line with these observations, our marine-sourced 6-bromoindole-3-acetonitrile also acts on intestinal barrier integrity and oxidative stress-related targets such as MPO, which further supports the potential of marine natural products as rich sources of agents for treating inflammatory bowel disease.

A cornerstone of UC pathology is the compromise of the intestinal barrier. Ni et al. suggested that restoring intestinal epithelium homeostasis could ameliorate colitis progression [30]. Our results demonstrate that 6-Bromoindole-3-acetonitrile promotes mucosal healing by increasing goblet cell numbers and enhancing *MUC2* expression, thereby restoring the protective mucus layer. The coordinated upregulation of key tight junction proteins (*Ocln*, *Cldn1*, *TJP1*) further confirms its role in fortifying the epithelial barrier [36].

Despite the promising findings, this study has several limitations. Notably, while we demonstrated significant transcriptional down-regulation of pyroptosis-related genes, the corresponding protein-level changes (e.g., the cleavage of *Caspase-1* and *GSDMD*) remain to be confirmed via Western Blotting. Future studies will focus on the protein-level dynamics and the specific molecular interactions between 6-bromoindole-3-acetonitrile and the *NLRP3* inflammasome complex to further elucidate its biochemical mechanism. Additionally, as an indole derivative, 6-bromoindole-3-acetonitrile may potentially interact with the gut microbiota or undergo microbial metabolism, aspects not addressed in this study. Furthermore, detailed pharmacokinetic evaluations are necessary to determine its metabolic stability and bioavailability before clinical translation.

In summary, our study identifies 6-Bromoindole-3-acetonitrile as a novel multi-target agent against preclinical experimental colitis. It exerts its beneficial effects by curtailing pyroptosis via *NLRP3* inflammasome inhibition, diminishing neutrophil influx and MPO activity, and restoring the structural and functional integrity of the intestinal barrier. These findings not only highlight the therapeutic potential of 6-bromoindole-3-acetonitrile but also support the feasibility of the strategy of mining marine natural products for drug candidates against complex inflammatory diseases like UC.

## Data Availability

The data presented in this study are openly available in [GEO] at [“https://www.ncbi.nlm.nih.gov/geo/ (accessed on 03 November 2025)”], reference number [GSE148794].

## Supplementary Materials

The following supporting information can be downloaded at: https://www.mdpi.com/article/10.3390/foods15101697/s1, Figure S1. Single-cell RNA sequencing analysis reveals changes in colitis-associated cell populations. Table S1. Scoring standards of the disease activity index (DAI) of the mice. Table S2. Primer sequences for quantitative real-time PCR in mouse tissue. Table S3. Primer sequences for quantitative real-time PCR in NCM460 epithelial cells. Table S4 Sequence and Name of 144 Compounds.
