# Supplementary material for: 6-Bromoindole-3-acetonitrile Attenuates DSS-Induced Colitis by Inhibiting Epithelial Cell Pyroptosis
Source: Foods. 2026 May 12;15(10):1697. doi: 10.3390/foods15101697 (PMC13205326; doi:10.3390/foods15101697)
Supplement: Supplementary file 1 [file foods-15-01697-s001.zip › foods-4202862-supplementary.pdf]

Table S1. Scoring standards of the disease activity index (DAI) of the mice.

Score	Loss of body weight	Stool consistency	Stool bleeding
0	<1%	Normal	Normal
1	1-5%	Soft granular stool	Occult blood
2	5-10%	Irregular loose and soft stool	A small amount of bloody stool
3	10-15%	Loose stool	A large amount of bloody stool
4	>15%	Diarrhea	Bloody stool

Table S2. Primer sequences for quantitative real-time PCR in mouse tissue.

Gene	Forward sequence	Reverse sequence
Tjp1	GCCGCTAAGAGCACAGCAA	GCCCTCCTTTTAACACATCAGA
Muc2	AGGGCTCGGAACTCCAGAAA	CCAGGGAATCGGTAGACATCG
Ocln	TGAAAGTCCACCTCCTTACAGA	CCGGATAAAAAGAGTACGCTGG
Cldn1	TGCCCCAGTGGAAGATTTACT	CTTTGCGAAACGCAGGACAT
Cldn2	CAACTGGTGGGCTACATCCTA	ATCCAGAGGCCCTTGGAAAAG
IL-6	CTGCAAGAGACTTCCATCCAG	AGTGGTATAGACAGGTCTGTTGG
IL-10	CTTACTGACTGGCATGAGGATCA	GCAGCTCTAGGAGCATGTGG
Il1β	GAAATGCCACCTTTTGACAGTG	TGGATGCTCTCATCAGGACAG
IL-17	TCAGCGTGTCCAAACACTGAG	CGCCAAGGGAGTTAAAGACTT
Tnf	CAGGCGGTGCCTATGTCTC	CGATCACCCCGAAGTTCAGTAG
IL-23	CAGCAGCTCTCTCGGAATCTC	TGGATACGGGGCACATTATTTTT
IL-18	GTGAACCCCAGACCAGACTG	CCTGGAACACGTTTCTGAAAGA
Casp1	ACAAGGCACGGGACCTATG	TCCCAGTCAGTCCTGGAAATG
Nlrp3	ATTACCCGCCCGAGAAAGG	CATGAGTGTGGCTAGATCCAAG
Pycard	GACAGTGCAACTGCGAGAAG	CGACTCCAGATAGTAGCTGACAA
Rela	TGCGATTCCGCTATAAATGCG	ACAAGTTCATGTGGATGAGGC

Table S3. Primer sequences for quantitative real-time PCR in NCM460 epithelial cells.

Gene	Forward sequence	Reverse sequence
TJP1	CAACATACAGTGACGCTTCACA	CACTATTGACGTTTCCCCACTC
MUC2	GAGGGCAGAACCCGAAACC	GGCGAAGTTGTAGTCGCAGAG
OCLN	ACAAGCGGTTTTATCCAGAGTC	GTCATCCACAGGCGAAGTTAAT
CLDN1	AGCTGCAAAATGTACGACTCG	GGAGACCACCATTAGGGCTC
CLDN2	GCCTCTGGATGGAATGTGCC	GCTACCGCCACTCTGTCTTTG
IL-6	ACTCACCTCTTCAGAACGAATTG	CCATCTTTGGAAGGTTCAGGTTG
IL-10	CGAGCGGACCTACTGTCCTA	GCCCAGTCAAGTGTCAGGTG
IL1β	ATGATGGCTTATTACAGTGGCAA	GTCGGAGATTCGTAGCTGGA
IL-17	TCCCACGAAATCCAGGATGC	GGATGTTCAGGTTGACCATCAC

TNF	CCTCTCTCTAATCAGCCCTCTG	GAGGACCTGGGAGTAGATGAG
IL-23	CTCAGGGACAACAGTCAGTTC	ACAGGGCTATCAGGGAGCA
IL-18	TCTTCATTGACCAAGGAAATCG G	TCCGGGGTGCATTATCTCTAC
CASP1	AGGCACGAGTAACAAGCTCAC	ATGAGGACATAACCAGCCACC
NLRP3	GATCTTCGCTGCGATCAACAG	CGTGCATTATCTGAACCCCAC
PYCAR D	TGGATGCTCTGTACGGGAAG	CCAGGCTGGTGTGAAACTGAA
RELA	ATGTGGAGATCATTGAGCAGC	CCTGGTCCTGTGTAGCCATT

Table S4. Sequence and Name of 144 Compounds.

Number	Name
1.	α-Vitamin E
2.	4-Hydroxybenzoic acid
3.	Palmitic acid
4.	Oxysophocarpin
5.	Spicalisporic Acid
6.	Formononetin
7.	Apigenin
8.	Trigonelline
9.	Lupeol
10.	Beta-Sitosterol
11.	PHYTOL
12.	Ursonic Acid
13.	Verbascoside
14.	{6}-Gingerol
15.	Isoacteoside
16.	Acacetin
17.	p-Hydroxybenzal dehyde
18.	Homogentisic acid
19.	Myristic acid
20.	L-Glutamic acid
21.	Stearic acid
22.	Vicenin 2
23.	Thymine
24.	Thymidine
25.	Heptadecanoic acid
26.	H-D-Trp-OH
27.	Pyrrole-2-caeboxylic acid
28.	N-Methylnicotina amide
29.	Aurantiamide
30.	Fucosterol
31.	Benzopinacole
32.	β-Cyclocitral
33.	(±)-Catechin

Number	Name
34.	α-Terpineol
35.	N-PENTADECANE
36.	Cyclo(Ile-Ala)
37.	Aurantiamide acetate
38.	B-Amyrin
39.	1-Hexanol
40.	trans,trans-2,4-Decadienal
41.	Pristane
42.	Methyl mycophenolate
43.	2,5-Dihydroxybenzaldehyde
44.	Allocholic acid
45.	Deoxylapachol
46.	Eicosane
47.	2-Chlorobenzene-1,3,5-triol
48.	2,4-dibroMobenzene-1,3,5-triol
49.	2-Pentadecanone
50.	2,4,6-tribromobenzene-1,3,5-triol
51.	1,2,3,4-Tetrahydronorharman-1-one
52.	4,6-Dlbromoindole
53.	7-Chlorokynureni acid
54.	3-bromopyrrole
55.	3-Chloro-6-Bromo Indole
56.	9H-Carbazole，1-methyl-carboxylic acid
57.	Ethyl6-bromo-1H-indole-3-carboxylate
**58.**	**6-Bromoindole-3-acetonitrile acid**
59.	6-Chloro-2,4-dibromophenol
60.	3-Amino-5-Hydroxybenzoic Acid
61.	2,4,6-Triphenylanill
62.	methyl 5-bromo-1H-pyrrole-2-carboxylate
63.	Methyl 6-bromo-1H-indole-3-carboxylate
64.	Sodium N-Octadecylsulfonate
65.	4-Amino-5-Bromopyrrolo[2,3-D]Pyrimidine
66.	1,3,5-Trimethyl-1H-Pyrazole-4-Carboxylic Acid
67.	6-Broomoindole-3-carbaxylie acid
68.	7-Bromoquinazoline-2,4(1H,3H)-Dione
69.	Methyl 5-bromo-1H-indole-3-carboxylate
70.	2,3-Dibromomaleni acid
71.	3-Bromo-4-Methoxyphenylacetic Acid
72.	4-Hydroxy-3-Nitrobenzyl Alcohol
73.	AC-PHE-OME
74.	2,7-Dibromo-9H-Carbazole
75.	Methy1 4-bromopyrrole--2-carboxylate
76.	9H-Carbazole,3,6-diiodo
77.	2,6-Dibromophenol
78.	Clorophene

Number	Name
79.	3-Bromobenzoic acid
80.	3,6-Dibromocarbazol
81.	2,,4,5-Tribromoimidazol
82.	2,4,6-Tribromophenol
83.	1,8-Diazabicyclo[5.4-0]undec-7-ene
84.	N-methyl-1H-indole-2-carbaldehyde
85.	ochracin
86.	2-Methyladenosin
87.	7-Cyano-7-deazaguanine
88.	1,3-Diphemethylure
89.	4,5-Dibromo-1H-Pyrrole-2-carbaldehyde
90.	3-chloro-1H-indole
91.	2-(3,5-dibromo-4-methoxyphenyl)acetic acid
92.	2-Isobutoxyanili
93.	Pseudane V
94.	5,6-dibromo-1H-indole-3- carbaldehyde
95.	2-(4-methoxyphenyl)-N-methyl-2-oxoacetamide
96.	Methyl 3-bromo-4,5-dihydroxybenzoate
97.	Mukanadin C
98.	3,3′-Bi[1H-indole]
99.	(+/-)-Longmide
100.	1H-Pyrrole-2-carboxylie acid,4,5-dibromo
101.	Cyclo(L-Phe-trans-4-hydroxy-L-Pro)
102.	2,6-Dibromo-4-(hydroxymethyl)phenol
103.	2-Pyrimidinamine,4-(5-bromo-1H-indol-3-yl)-amine
104.	1,7-dimethyl-1H-indole-3-carbaldehyde
105.	5-[(1H-indole-3-yl)methylidene]imidazolidine-2,4-dione
106.	3,4,-Diphenyl-5H-furan-2-one
107.	Methyl 5-hydroxy-1H-indole-3-carboxylate
108.	6-Hydroxyisatin
109.	6-Bromo-1H-indole-3-acetic acid methyl ester
110.	Pygmaniline B
111.	(3E)-4-(1H-Indol-3-yl)but-3-en-2-one
112.	Indole-3-glyoxylamide
113.	1-(2-hydroxyethyl)thiophene
114.	Cyclo(Pro-Val)
115.	Cyclo(Pro-Leu)
116.	α-Conotoxin GI acetate
117.	5,6-dibbromo-1H-indole-3-**c**arbaldehyde
118.	2-broMobenzene-1,3,5-triol
119.	3-Indoleacrylic acid
120.	Spongosine
121.	Thiamine monochloride
122.	1-beta-D-Arabinofuranos
123.	Ser-Ala-alloresact

Number	Name
124.	Uracil
125.	Oleanolic Acid
126.	Tramiprosate
127.	Quercetin
128.	Azelaic acid
129.	(-)-Epicatechin
130.	Gamma-Linolenic acid
131.	Arachidic acid
132.	Eicosapentaenoic Acid
133.	Arachidonic acid
134.	Fucoxanthin
135.	Pyropheophorbide-a
136.	Squalane
137.	Citric acid
138.	Cedrol
139.	Farnesylacetone
140.	1-0-Hexadecyl-Rac-Glycerol
141.	L-Aspartic acid
142.	Riboflavin phosphate Sodium
143.	Γ-Aminobutyric Acid
144.	Taurine

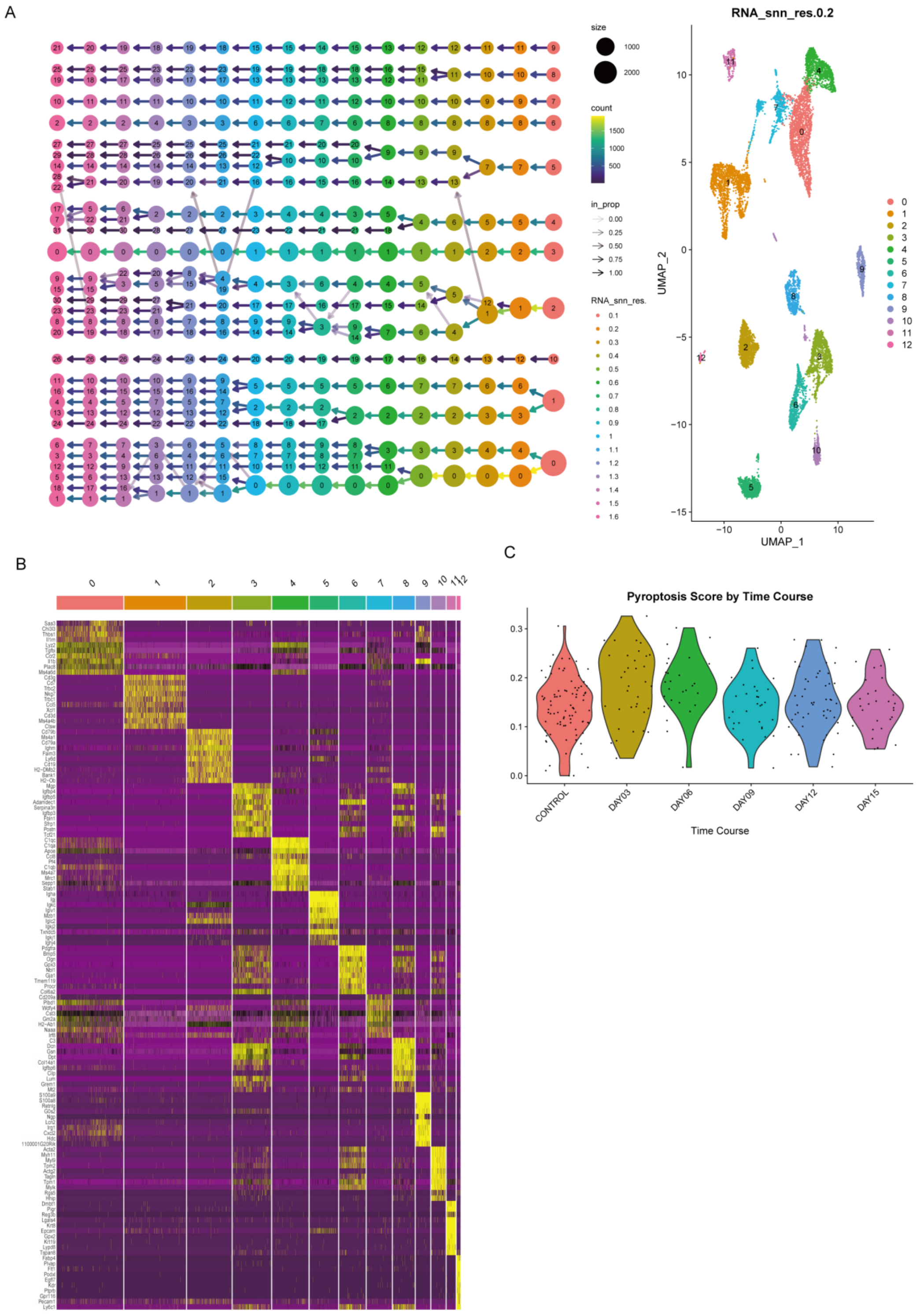

Supplementary Figure S1. Single-cell RNA sequencing analysis reveals changes in colitis-associated cell populations. (A) Clustree analysis showing the number of cell populations at different resolutions and the UMAP plot at a resolution of 0.2; (B) Top 10 highly variable genes in each cluster; (C) Scatter plot of pyroptosis scores in mice from the normal group and model groups at different time points.
